# 2.5D Multi-View Gait Recognition Based on Point Cloud Registration

**DOI:** 10.3390/s140406124

**Published:** 2014-03-28

**Authors:** Jin Tang, Jian Luo, Tardi Tjahjadi, Yan Gao

**Affiliations:** 1 School of Information Science and Engineering, Central South University, Changsha 410083, China; E-Mails: tjin@csu.edu.cn (J.T.); delphifx@csu.edu.cn (J.L.); 2 School of Engineering, University of Warwick, Gibbet Hill Road, Coventry CV4 7AL, UK; E-Mail: t.tjahjadi@warwick.ac.uk

**Keywords:** gait, person identification, 2.5D modeling, point cloud registration

## Abstract

This paper presents a method for modeling a 2.5-dimensional (2.5D) human body and extracting the gait features for identifying the human subject. To achieve view-invariant gait recognition, a multi-view synthesizing method based on point cloud registration (MVSM) to generate multi-view training galleries is proposed. The concept of a density and curvature-based Color Gait Curvature Image is introduced to map 2.5D data onto a 2D space to enable data dimension reduction by discrete cosine transform and 2D principle component analysis. Gait recognition is achieved via a 2.5D view-invariant gait recognition method based on point cloud registration. Experimental results on the in-house database captured by a Microsoft Kinect camera show a significant performance gain when using MVSM.

## 1. Introduction

Gait recognition is a means of using the behavioral biometrics of gait to identify a human subject. Gait is difficult to disguise and can be easily observed in low-resolution video sequences. The need for a means for counter-terrorism, security and medical-related subject behavior analysis makes accurate modeling of human gait and effective extraction of gait signatures for view-invariant subject identification have significant theoretical and practical value. For example Chowdhury and Tjahjadi [1] proposed a gait recognition method that combines spatio-temporal motion characteristics, statistical and physical parameters of a human subject to achieve robustness and high accuracy in subject identification.

In surveillance applications, most of the challenging factors that affect existing gait recognition systems [2], e.g., variation in human walking posture for different camera views, make the performance of a gait recognition method that is designed to operate on a particular camera view degrade significantly for other views. Furthermore, for gait recognition to be used in surveillance applications, it is impractical to use many cameras to achieve multi-view gait recognition. Thus, achieving view-invariant gait recognition has become a major challenge.

There are several approaches to view-invariant gait recognition. One approach is to reconstruct 3-dimensional (3D) gait models using a calibrated multi-camera system and extract 3D gait features. Shakhnarovich *et al.* [3] explored the use of an image-based visual hull to reconstruct the 3D model and rotate the model to realize view-invariant gait recognition. Gu *et al.* [4] proposed viewpoint-free gait recognition from recovered 3D human joints. Sivapalan *et al.* [5] proposed the use of a 3D voxel model derived from multi-view silhouette images. However all current examples of 3D modeling of the human body are mostly based on images from multiple cameras. Due to the need for multiple equipment and the increased complexity of the resulting recognition algorithm, such an approach is usually only feasible under laboratory conditions. In addition, although radar (e.g., laser radar) can also be used for the 3D modeling, the resolution of the resulting model is low.

The second approach is to use view transformation model (VTM) to achieve multi-view gait recognition. VTM transforms gait features from different views onto the same view. The VTM is constructed by decomposing a matrix comprising features from different views and of different subjects into subject-independent matrix and view-independent matrix. Makihara *et al.* [6] used VTM to transform gallery features onto the same view for multi-view gait recognition. Muramatsu *et al.* [7] proposed an arbitrary gait view transformation scheme using 3D gait database and VTM method. Kusakunniran *et al.* [8,9] developed the VTM model by using correlated motion regression and multi-layer perceptron. Although the VTM gait recognition approach demonstrates the advantages of multi-view gait recognition, it requires multiple-view images to generate VTM. Furthermore, the model accuracy is determined by the number of multi-view gaits used in the VTM construction.

The third approach is to use a multi-view fusion classifying method. By fusing gait classification from multi-view data captured by multiple cameras, view-invariant gait recognition is realized. For example, Nizami *et al.* [10] explored the use of Extreme Learning Machine (ELM) multiclass classifier for classification, and the results are fused at score level subject to some fusion rules to realize the view-independent gait recognition. However, the ELM based system does not address the problem of using multi-view images. To address this problem, Jean *et al.* [11] proposes an approach to compute view-normalized body part trajectories. The normalized trajectories are extracted as view-invariant gait feature for gait recognition. However human gait information cannot be fully represented using only trajectories of head and feet. Thus, when the gait views are significantly changed or self-occlusion is encountered, the method performs poorly.

To address the above-mentioned problems, in this paper we propose the use of a single Kinect camera to obtain point cloud data of a human body and construct 2.5D voxel gait model that includes only one-side surface portion of the human body. A point cloud registration method is proposed to synthesize multi-view gait features using two reconstructed gait models from two different views. Dense point cloud and view-invariant Gaussian curvature are extracted to represent the gait features. The 2.5D data is mapped onto the 2D space, and Gaussian curvature based gait color images are used to facilitate the gait feature extraction, classification and identification of the human subject.

This paper is organized as follows: Section 2 presents the construction of 2.5D gait voxel model using a Kinect device with point cloud data simplification. Section 3 presents point cloud registration for multi-view 2.5D gait voxel model. Section 4 introduces the extraction of 2.5D gait features and the multi-view gait recognition method of the proposed gait recognition system. Section 5 presents our experimental results and Section 6 concludes the paper.

## 2. Preliminary Steps

### 2.1. Construction of 2.5D Gait Voxel Model

**Figure 1.**
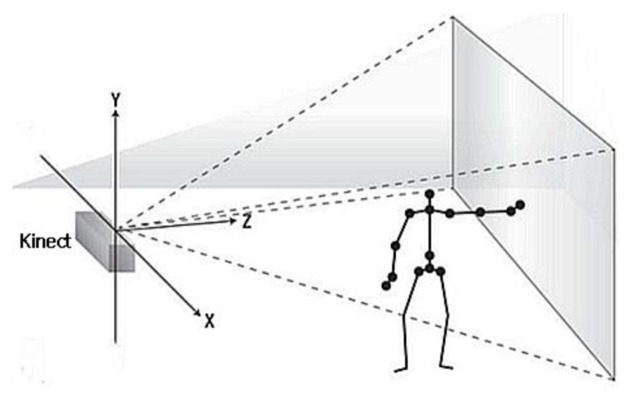
World coordinates of the Kinect sensor-based system.

2.5D data that contains depth information is used to construct gait surface voxel model, and a Kinect is used to capture the 2.5D data which is a simplified 3D (*x,y,z*) surface representation (Figure 1). 2.5D data contains at most one depth value d(*x,y*) which denotes the distance between the RGB image pixel (*x,y*) of a point on the body surface and the Kinect. 2.5D is a suitable trade-off solution between 2D and 3D approaches. It is restricted to a given viewpoint that is called 2.5D information [12].

As a 3D measuring device, Kinect comprises an IR pattern projector and an IR camera. It can output three different images: IR image, RGB image and Depth image. The 2.5D data of the depth image and RGB image are used to construct a 3D voxel model for a given viewpoint by calculating all the 3D points from the measurement (*x,y,d*) in the depth image. 3D point cloud data are calculated using the Kinect geometrical model [13], *i.e.*:

(1) $$\left[\begin{array}{l} X\\ Y\\ Z \end{array}\right]=\frac{1}{c_{1}d+c_{0}}dis^{-1}\left(K^{-1}\left[\begin{array}{c} x+u_{0}\\ y+v_{0}\\ 1 \end{array}\right],k\right)$$

where *d* is depth value along the *z*-axis, *c*_1_ and *c*_0_ are parameters of the model, *u*_0_ and *v*_0_ are respectively the shifted parameters of IR and depth images, *dis* is distortion function, *k* is distortion parameter of the Kinect IR camera and *K* is the IR camera calibration matrix.

**Figure 2.**
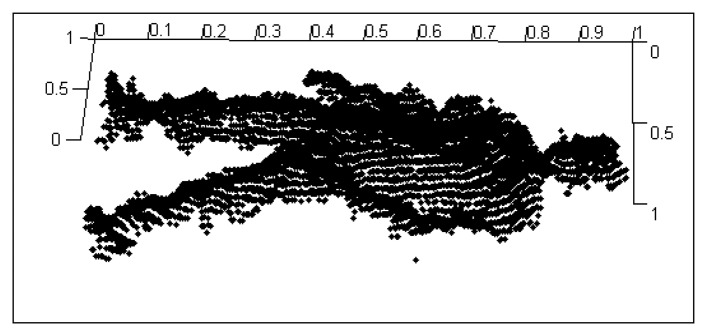
The normalized point cloud data of human body.

Before constructing the 2.5D gait point model, gait silhouettes are extracted from the depth image by foreground substraction and frame difference methods [14]. The gait silhouettes and RGB images are then used to calculate all the 3D point cloud data for the gait using Equation (1). The 3D point cloud gait model is constructed for a given viewpoint by normanizing all the gait point cloud data to 3D space. Since only a single Kinect depth camera is used, the gait point cloud data includes only one side surface portion of the human body as shown in Figure 2. We call it a 2.5D voxel model.

### 2.2. Point Cloud Data Simplification for Gait Voxel Model

Since the point cloud data is large, it is simplified while preserving its features. This is achieved by using curvature features of the point cloud by Hausdorff distance [15]. A bounding box method is first used to derive the relationship between a point cloud data *P* and its *K* nearest neighbors. Denote the two principal curvatures of *P* and one its neighboring points respectively as $\left\{K_{1}^{P},K_{2}^{P}\right\}$ and $\left\{K_{1}^{Q},K_{2}^{Q}\right\}$. The Hausdorff distance *H* of the two data sets is:

(2) $$H=\underset{i=1,2}{\max}\underset{j=1,2}{\min}\left(\frac{\left\|K_{i}^{P}-K_{j}^{Q}\right\|}{\left\|K_{i}^{P}\right\|+\left\|K_{j}^{Q}\right\|}\right)$$

**Figure 3.**
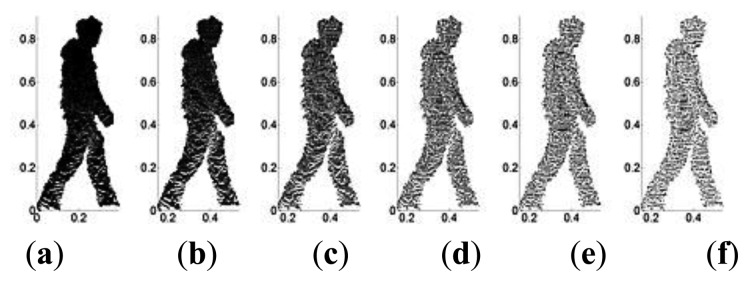
Simplification of point cloud data: (**a**) raw data; (**b**) *ε* = 10^−6^; (**c**) *ε* = 10^−5^; (**d**) *ε* = 10^−4^; (**e**) *ε* = 10^−3^; and (**f**) *ε* = 10^−2^.

The Hausdorff distance is defined for *P* as *H^P^* = max(*H^Q^*),*Q* = 1,2,…*k*. By calculating the Hausdorff distance of every point within the bounding box, a threshold *ε* is selected to remove the less important point cloud data, and thus complete the data point simplification as shown in Figure 3. The choice of the threshold *ε* directly influences the efficiency of the simplification and the computational cost of the algorithm. A bigger *ε* will reduce computational cost but less simplifying efficiency, while a smaller *ε* has the opposite effect. We conducted experiments to determine the optimum *ε* value. 2.5D gait voxel models are selected for point cloud simplifying experiment and *ε* is set to 10^−6^, 10^−5^, 10^−4^, 10^−3^ and 10^−2^.

Figure 3a shows a raw gait point cloud data (including 25,862 point cloud data) before simplification, and Figure 3b–f is the results after simplification with 13,286, 8,392, 6,381, 4,592 and 2,392 point cloud data, respectively. The computational times are 518, 432, 327, 273 and 228 ms, respectively. From the experiment results, we set *ε* = 10^−4^ with mean computational time and sufficient simplification.

## 3. Point Cloud Registration for Multi-View 2.5D Gait Voxel Models

### 3.1. Overview

**Figure 4.**
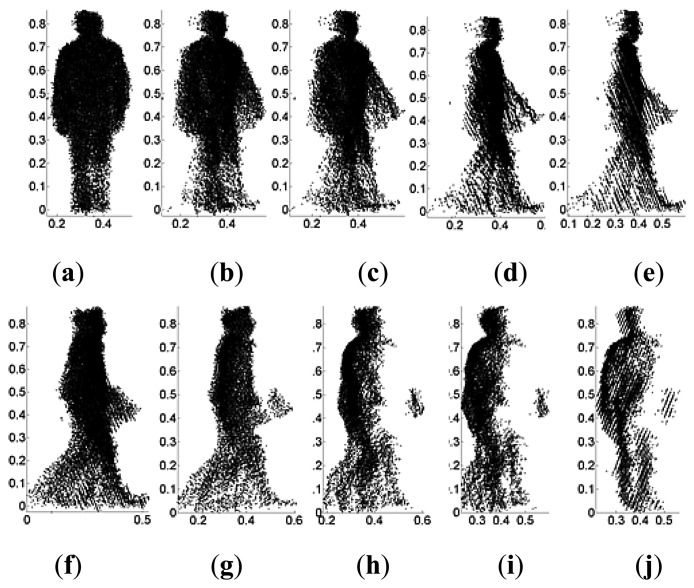
Self-occlusion caused by rotating 2.5D gait model: (**a**–**e**) 0° view rotated counterclockwise; and (**f**–**j**) 90° view rotated clockwise.

Since the 2.5D gait voxel model is constructed from data captured using a single Kinect camera, view-invariant gait recognition cannot be realized by just rotating the model to obtain gait features for different views due to self-occlusion as illustrated in Figure 4.

In order to realize multi-view gait recognition and overcome the self-occlusion problem with 2.5D gait models, a point cloud registration method is proposed to synthesize different view gait features. And two training galleries with *θ*_min_ and *θ*_max_ views are used. Let *P*_min_ be the 2.5D gait point cloud data for *θ*_min_ view and *P*_max_ for *θ*_max_ view where *P*_min_ ∩ *P*_max_ ≠ Φ. *P*_min_ is registered with *P*_max_, and the new *β* view point cloud data after registration is represented by:

(3) $$S_{\beta }=F_{\mathrm{reg}}(P_{\min},P_{\max})=(P_{\min}\cdot R_{\left(\theta_{\min}\to \beta \right)}+T_{1})\cup (P_{\max}\cdot R_{\left(\theta_{\max}\to \beta \right)}+T_{2})$$

If *â* is set to *θ*_min_ then:

(4) $$S_{\beta }=S_{\theta_{\min}}=P_{\min}\cup (P_{\max}\cdot R_{\left(\theta_{\max}\to \theta_{\min}\right)}+T)$$

where *R*_(_*_θ_*_max →_*_β_*_)_ denotes 3D rotation matrix from view *θ*_max_ to *â*, and *T* denotes the translation matrix. Based on the registered point cloud data *S_â_* with *â* view, the 2.5D gait data between *θ*_min_ and *θ*_max_ views can be determined without encountering any self-occlusion problems using:

(5) $$P_{\theta }=S_{\beta }\cdot R_{\left(\beta \to \theta \right)}$$

**Figure 5.**
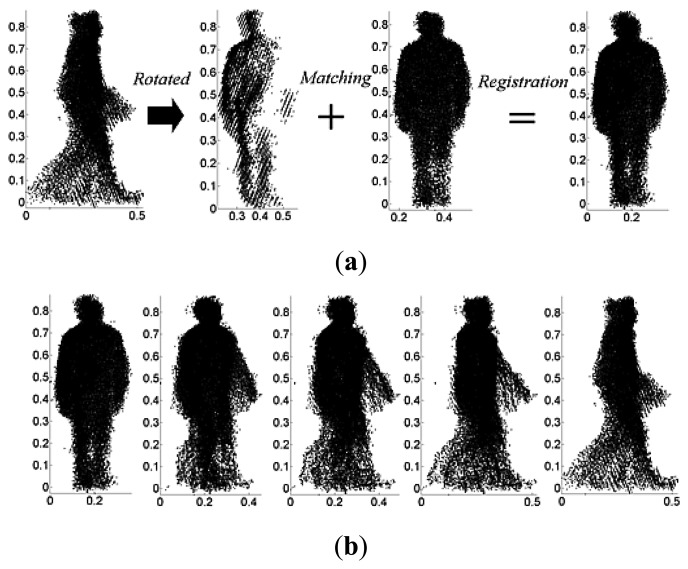
Self-occlusion problem overcome by point cloud registration method: (**a**) gait point cloud registration with *θ*_min_ = 0°, *θ*_max_ = 90° and *â* = 0°; and **(b**) view rotated from registered gait without self-occlusion.

The self-occlusion problem is addressed by the point cloud registration and 2.5D curvature features extraction method as illustrated in Figure 5. This is why in our approach only two different view galleries are needed for multi-view gait training and recognition.

### 3.2. Gait Point Cloud Alignment in a Cycle

**Figure 6.**
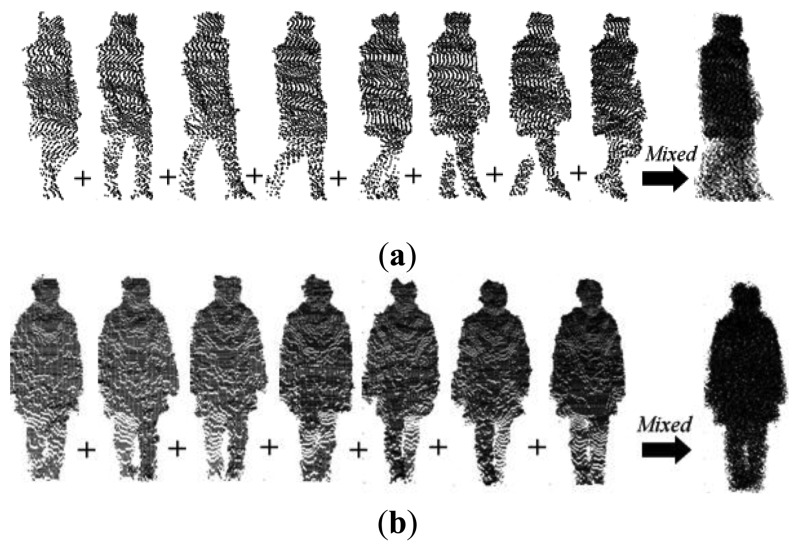
Gait point cloud alignment and mixed gait modeling in a cycle: (**a**) 0° gait view; and (**b**) 90° gait view.

**Figure 7.**
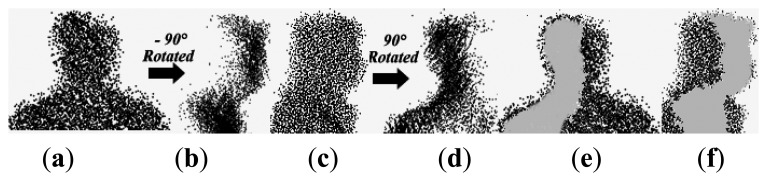
Mixed head point cloud: (**a**) 0° head view; (**b**) −90°rotated; (**c**) 90° head view; (**d**) 90° rotated; (**e**): overlapped areas of (a) and (d); and (**f**) overlapped areas of (b) and (c).

Gait features are represented by a complete cycle as shown in Figure 6. It can be seen that a gait cycle must include several dynamic frames. 2.5D gait models are reconstructed by data from corresponding frames. Therefore point cloud registration cannot be made directly between two random 2.5D models with different views and cycles. Most often, the number of frames in a gait cycle is different, e.g., in Figure 6 the sample gait with 0° has eight frames while the corresponding gait with 90° has only seven frames. It is thus difficult to directly register models between two different view cycles. In order to overcome these problems, only the head point cloud data as shown in Figure 7 is used to calculate the rotation matrix and translation matrix for registration. The resulting matrices are used in registration process between full gait models. The first step is to align the gait point cloud data in the same cycle by using the centroids of head point cloud data. The head information is used because it is static information when compared with legs and arms.

Assume that after gait phase estimation N frames in a gait cycle are extracted, and the corresponding 2.5D gait models are constructed. The 2.5D gait models are denoted as $P_{\theta }=\{p_{\theta }^{i},i=1\ldots N\}$. The extracted head models are denoted by $H_{\theta }=\{h_{\theta }^{i},i=1\ldots N\}$. The centroids of all head models are calculated as $\mu (H_{\theta })=\{\mu (h_{\theta }^{i}),i=1\ldots N\}$. In order to complete alignment, the first gait model is set as reference. The translation matrices that align with the first gait model are then calculated using $T^{i}=\mu (h_{\theta }^{i})-\mu (h_{\theta }^{1}),i=1\ldots N$. The final mixed 2.5D gait model after alignment is given by $M_{\theta }=\{\cup (p_{\theta }^{i}+T^{i}),i=1\ldots N\}$.

### 3.3. Gait Point Cloud Registration

After gait point cloud alignment in a cycle, a mixed 2.5D gait model is obtained from different gait models in a gait cycle, which represents the 2.5D gait features. Gait point cloud registration is then conducted between two mixed gait models of different views.

In order to complete point cloud registration between two 2.5D mixed gait models with *â* = *θ*_min_ in Equation (4), the rotation matrix *R*_(_*_θ_*_max→_*_θ_*_min)_ and translation matrix *T* need to be determined using iterative closest point algorithm (ICP) [16]. Let $H_{\min}^{\text{mix}}$ be the head point cloud in *θ*_min_ mixed training model and $L_{\min}=\{l_{\min}^{i}\in R^{3},1\leq i\leq n\}$represents the overlapped area of head point cloud data with *θ*_max_ view. $H_{\max}^{\text{mix}}$ denotes the head point cloud data in *θ*_max_ mixed training model, and $L_{\max}=\{l_{\max}^{i}\in R^{3},1\leq i\leq m\}$ represents the overlap area of head point cloud data with *θ*_min_ view.

There are common areas of two different view head surfaces as shown in Figure 7. The accurate detection of the overlapped region will aid the gait point cloud registration. The optimization process that determines the rotation matrix *R*_(_*_θ_*_max→_*_θ_*_min)_ and translation matrix *T* is a nonlinear least squares optimization [17], *i.e.*:

(6) $$\min E(g)=\sum_{i=1}^{\min(\text{m},\text{n})}{\left\|gl_{\max}^{i}-l_{\min}^{i}\right\|}^{2}$$

Let *_g͂_* be the optimum solution, then the point cloud set $L_{\max}^{\prime }=\{l_{\max}^{\prime_{i}}=\tilde{g}l_{\max}^{i},1\leq i\leq n\}$ has the same centroid with *L*_min_, and let $\mu_{\left(L_{\min}\right)}=\mu_{\left(L_{\max}^{\prime }\right)}$. We then calculate the centroid of *L*_min_ and *L*_max_ as *μ*_(_*L*_min)_ and *μ*_(_*L*_max)_, where $\mu_{\left(L_{\max}^{\prime }\right)}=\tilde{g}\mu_{\left(L_{\max}\right)}$. Let $d_{i}=l_{\max}^{i}-\mu_{\left(L_{\max}\right)}$, $d_{i}^{\prime }=l_{\min}^{i}-\mu_{\left(L_{\min}\right)}$, Equation (6) then becomes:

(7) $$\min E(R)=\sum_{i=1}^{\min(\text{m},\text{n})}{\left\|Rd_{i}-{d^{\prime }}_{i}\right\|}^{2}$$

The optimization then decomposes into determining the rotated matrix R, and calculating the translation matrix *T* = *μ*_(_*L*_min)_ − *μ*_(_*L*_max)_ · Singular value decomposition (SVD) method is used to calculate R as follows. First, the covariance matrix D between *L*_min_ and *L*_max_ is calculated as:

(8) $$D=\sum_{i=1}^{\min(\text{m},\text{n})}d_{i}d_{i}^{\prime T}$$

The matrix *D* is then decomposed by SVD, and let *D* = *UVV^T^*, *X* = *VU^T^* and:

(9) $$A=\{\begin{array}{ll} X & \det(U)\det(V)\geq 0\\ \mathrm{diag}(1,1,-1) & \det(U)\det(V)<0 \end{array}$$

The determinant of *U* is denoted by det(*U*), and *diag*(1,1,−1) denotes the 3 × 3 matrix that has diagonal values of 1, 1, −1. If *rank(D)* ≥ 2, then *R* and *T* are respectively given by:

(10) $$R=UAV^{T}$$

(11) $$T=\mu_{\left(L_{\min}\right)}-R\mu_{\left(L_{\max}\right)}$$

*L*_max_ point could data is then transformed using *R* and *T*. After transformation a new point cloud data set $L_{\max}^{\prime }$is obtained by $L_{\max}^{\prime }=R\cdot L_{\max}+T$.

We then repeat the previous steps to calculate new *R* and *T* by conducting iterative transformation until the square distance error $e={\overset{\min(\text{m},\text{n})}{\underset{i=1}{\text{∑}}}\left\|R_{k}l_{\max}^{i}+T_{k}-l_{\min}^{i}\right\|}^{2}$ satisfies the smallest requirements. The matrices R and T are then used to construct the final registration gait model:

(12) $$S_{\beta }=S_{\theta_{\min}}=M_{\theta_{\min}}\cup (M_{\theta_{\max}}\cdot R_{\left(\theta_{\max}\to \theta_{\min}\right)}+T)$$

The key of the algorithm is to detect the overlapped region of two views accurately and construct the covariance matrix. The method to determine the matching point set is as follows. First, we calculate the Gaussian curvature *K* of the point cloud data for the head part of 2.5D gait models $H_{\max}^{\text{mix}}$ and $H_{\min}^{\text{mix}}$. Gaussian curvature is invariant to the affine transformation and is used as the basis of the matching. The similar point cloud data are then searched between $H_{\min}^{\text{mix}}$ and $H_{\max}^{\text{mix}}$. Let $\forall q_{i}\in H_{\min}^{\text{mix}}$, $p_{i}\in H_{\max}^{\text{mix}}$, and we define the curvature distance between two point cloud data as *Dis*(*q_i_,p_i_*) = |*K*(*q_i_*) − *K*(*p_i_*)|. The similar point cloud data is then determined and forms the matching point set:

(13) $$S=\{(p_{i},q_{i})|q_{i}\in H_{\min}^{\mathrm{mix}},p_{i}\in H_{\max}^{\mathrm{mix}},\delta <\text{Dis}(p_{i},q_{i})<ɛ\}$$

where $ɛ=\frac{1}{2}(\max\text{Dis}(q_{i},p_{i})+\min\text{Dis}(q_{i},p_{i}))$ and $\delta =\frac{1}{N}\sum_{i=1}^{n}\left|K(q_{i})\right|$.

Since one point cloud data may be similar to many point cloud data in another point cloud data set, one to one correspondence analysis are performed by matching similar triangles. We first select three points in *S* from $H_{\theta_{\min}}^{\max}$, and search the most similar three points in *S* from $H_{\max}^{\text{mix}}$ using similar triangles, where the distance between two triangles is

(14) $$T_{\mathrm{dis}}=\frac{1}{3}\times \sum_{1\leq i<j\leq 3}\frac{\left|\left|p_{i}p_{j}\right|-\left|q_{i}q_{j}\right|\right|}{\left|p_{i}p_{j}\right|+\left|q_{i}q_{j}\right|}$$

The points with the smallest distance are selected as matching cloud point. When all the points are matched, the covariance matrix *D* is determined for computing the matrices *R* and *T*. to achieve two view gait point cloud model registration as Equation (12). The registration model can then be rotated to obtain gait features for different views to achieve view-invariant gait recognition using Equation (5).

## 4. 2.5D Gait Features Extraction and Multi-View Gait Recognition

### 4.1. 2.5D Gait Features Extraction

The density of point cloud is utilized to extract silhouette data of the human subject, and Gaussian curvature and mean curvature [18] are used to extract 2.5D gait features. The color gait curvature image (CGCI) for gait recognition is formed by mapping the 2.5D gait features to a color gait image.

#### 4.1.1. Point Cloud Density, Gaussian Curvature, Mean Curvature and CGCI

**Figure 8.**
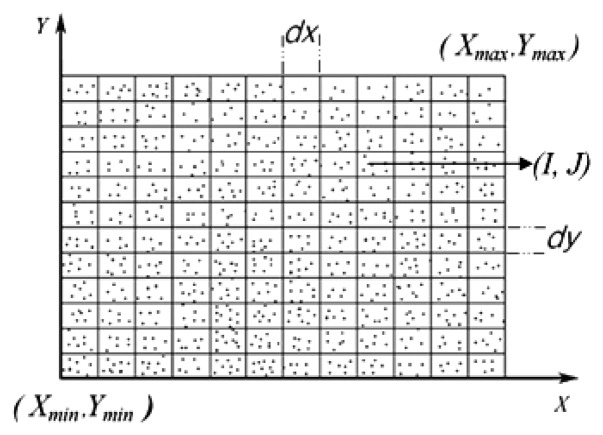
Projection of normalized point cloud data onto blocks.

The normalized point cloud data is first projected onto the XY plane into *N_I_* × *N_J_* blocks as shown in Figure 8, where *dx* and *dy* are respectively the horizontal and vertical sampling intervals. Each point cloud data is located in the corresponding block (*I,J*), and each block may have several point cloud data. In order to extract the silhouette information of the gait, the density of point cloud in each block (*I,J*) is calculated first, denoted by *Density*(*I,J*).

The curvature information is extracted next. Since only a single Kinect camera is used, the point cloud data includes only one side surface portion of the human body, and the surface point set is denoted by *S* = [*x,y,z*], (*x,y*) ∈ *D*, where *D* is the projected grid from 2.5D gait surface onto the XY plane. Before extracting the curvature information, the mean value of z are calculated for all point cloud data located in the same block (*I,J*), denoted by *z_mean_*_(I,J)_. The discrete surface point set is then obtained *S_dis_* = [*I,J,z_mean_*_(I,J)_].

Gaussian curvature and mean curvature are view-invariant under certain class of geometric transformation including rotation, scaling and shearing [18]. Gaussian curvature *K* and mean curvature H are then computed through the discrete surface point set *S_dis_* by the method in [19], respectively denoted as *K*_(_*_I_*,*_J_*_)_ and *H*_(_*_I_*,*_J_*_)_ for each block (*I,J*).

**Figure 9.**
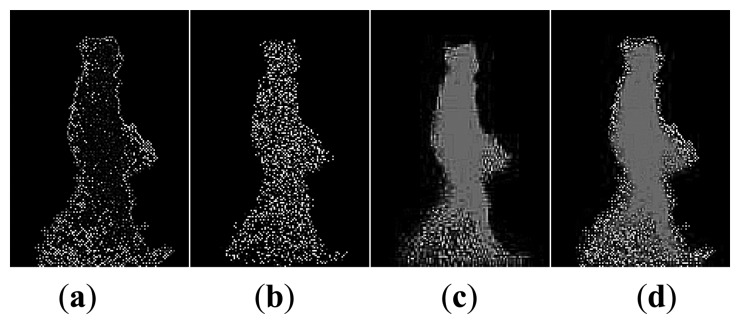
Curvature based 2D color gait feature image (CGCI): (**a**) R component; (**b**) G component; (**c**) B component; and (**d**) RGB image.

The curvatures *K* and *H* in 2.5D space are normalized to the range [0, 2^16^], and the density of point cloud is also normalized to [0, 255]. They are then projected onto 2D RGB space to facilitate the gait feature extraction, classification and identification of the human subject. A pseudo 2D RGB color image is obtained by mapping *K*_(_*_I_*,*_J_*_)_ and *H*_(_*_I_*,*_J_*_)_ of each block to the red (R), green (G) and blue (B) components of the image. The R and G components of the image respectively denote the most significant 8 bit value and the low significant 8 bit values of the curvature. The B component of the image denotes the density of gait point cloud data that indicates the average silhouette information of human subject. The size of the resulting RGB image, *i.e.*, CGCI, is *N_I_* × *N_J_* and is shown in Figure 9. The final CGCI is denoted as *A* = {*R*(*I,J*),*G*(*I,J*),*B*(*I,J*)} ∈ *R^NI^*^×^*^NJ^*.

### 4.2. Multi-View Gait Recognition

**Figure 10.**
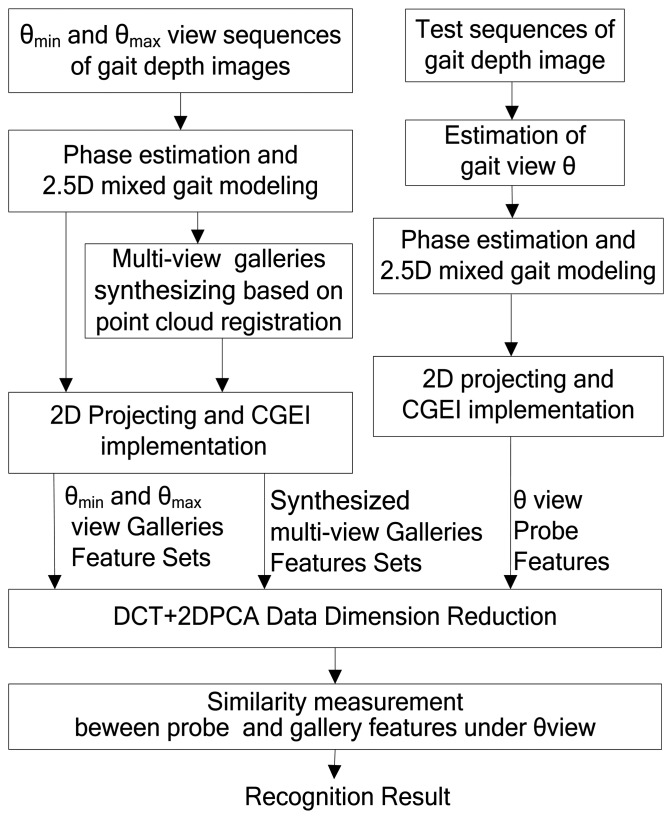
2.5D view-invariant gait recognition method based on point cloud registration.

To realize multi-view gait recognition with a single Kinect depth camera, two standard reference gait views *θ*_min_ and *θ*_max_ are needed as a training gait set. Figure 10 shows the 2.5D view-invariant gait recognition method based on point cloud registration using CGCI in detail. In paper, 0° and 90° view of gait depth images are selected as standard reference training gait sets.

#### 4.2.1. Estimation of Phase and View Angles for 2.5D Gait

In this paper, the gait phase is estimated using the width information of two legs in motion by silhouette depth images. The view angle of a test sample also needs to be estimated to enable it to be transformed or compared with the corresponding training samples. However it is difficult to obtain a reliable estimation from 2D images. Several methods have been proposed to estimate the gait angles. The method in [11] uses body part trajectories during walk to realize gait view normalization. This method does not work well when the difference in view angles is large. The method in [20] uses the regression models learned from training gait database. The estimation is poor when there is self-occlusion. The method in [21] uses perspective projection to estimate the walking angle in the 3D world from a video sequence of a planar scene. This method requires camera calibration and also performs poor when the difference in view angles is large.

**Figure 11.**
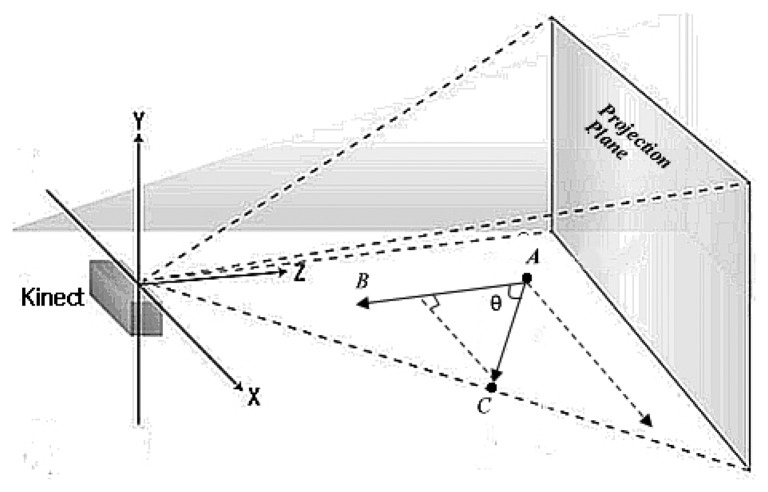
Estimation of view angles for 2.5D gait.

A reliable method is proposed for view angle estimation in 2.5D space from video sequences. The subject is assumed to be walking along a straight line *AC* and line *AB* is parallel to the *Z* axis as shown in Figure 11. First, *Nth* and (*N* + *k*)*th* depth image frames are selected in a gait cycle. The two selected depth images are then used to construct 2.5D voxel gait model in the world coordinate. Let (*x_i_,y_i_,z_i_*), *i* ∈ {1,2,…*N*} represent the point cloud data of gait where N denotes the total number of point cloud data. The centroids of the *Nth* and (*N* + *k*)*th* 2.5D gait model are then calculated, denoted by (*X*_1_*_C_*, *Y*_1_*_C_*, *Z*_1_*_C_*) and (*X_kC_*, *Y_kC_*, *Z_kC_*) respectively. The estimation azimuth angle θ is given by *Tanθ* = (*X_kC_* − *X*_1_*_C_*)/(*Z_kC_* − *Z*_1_*_C_*).

Table 1 shows the gait view angle estimation experiment results using 100 subjects for each subject for 0°, 15°, 30°, 45°, 60°, 75°, 90° views. The experimental results demonstrate that the proposed method is feasible.

#### 4.2.2. Multi-View Galleries Synthesizing

Before synthesizing multi-view galleries, two standard reference mixed gait models *M_θ_*_min_ and *M_θ_*_max_ must be constructed by the method in Section 3.2. This is followed by point cloud registration to form a new 2.5D mixed gait model *S_â_*. Multi-view galleries are then synthesized. It is based on rotating the new registered gait model *S_â_* in Δ*θ* step interval to obtain gait features for different view between two reference views. Let $P_{k}^{\theta }$ denotes the *θ* view synthesized 2.5D gait galleries, *i.e.*:

(15) $$P_{k}^{\theta }=S_{\beta }\cdot R_{\left(\beta \to \theta \right)},k=1\ldots N,\theta =0{}^{\circ},\Delta \theta ,2\Delta \theta ,\ldots ,90{}^{\circ}$$

where *N* represents the number of training objects. The density of point cloud data and 2.5D curvature features are then extracted from $P_{k}^{\theta }$. Finally gait CGCI features with different view from 0° to 90° are obtained denoted as $A_{k}^{\theta }\in R^{N_{I}\times N_{J}}$. *k* = 1…*N*.

#### 4.2.3. DCT and 2DPCA Based Gait Feature Dimension Reduction

**Figure 12.**
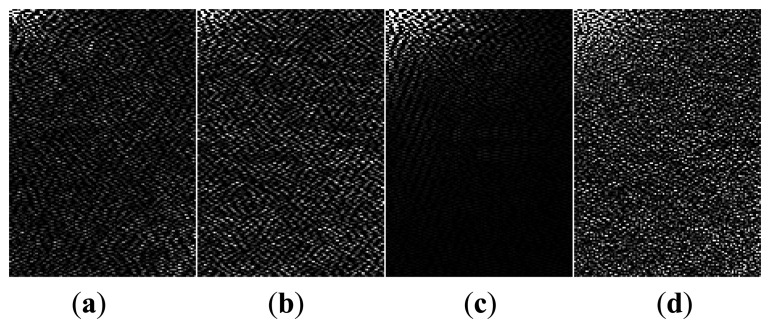
DCT transform of CGCI: (**a**–**d**) are respectively the DCTs of (a–d) in Figure 9.

The size of a CGCI is determined by the sampling intervals *dx* and *dy* (see Section 4.1). 2D discrete cosine transforms (2D-DCT) [22] and 2-dimensional Principle Component Analysis (2DPCA) is used to reduce the dimensionality of the gait feature space. DCT is applied to R, G and B components of the CGCI separately. The DCT coefficient matrices are then obtained with the same size as CGCI. The low frequency components containing the most important information of the image are concentrated in the upper left corner of the DCT matrix, while the high frequency components are distributed in the lower right corner as shown in Figure 12. Since the high frequency components are less important, we only need to retain the low frequency components when reconstructing the image.

A *m* × *m* matrix is used to extract the low frequency DCT coefficients in the upper left corner. The gait features extracted from DCT coefficients from *N* CGCIs with *α* view are then denoted by $A_{k}^{\alpha }\in R^{m\times m}$ *K* = 1…*N*, 2DPCA is then applied to further reduce the dimensions. Unlike PCA which involves vectors, 2DPCA deals with matrices corresponding to images, and uses a matrix to construct a covariance matrix [23]. After extracting multi-view CGCI-DCT gait features, the data dimensionality of gait features for each view is then conducted further reduction. Let CGCI-DCT feature matrices with N objects be denoted by:

(16) $$A_{k}^{\alpha }\in R^{m\times m},k=1\ldots N,\alpha =0{}^{\circ},\Delta \theta ,2\Delta \theta ,\ldots ,90{}^{\circ}$$

The mean matrix $\bar{A^{\alpha }}$ and covariance matrix *S^á^* of the N DCT matrices are then calculated. Eigenvalue decomposition is then performed on *S^á^*, *i.e.*:

(17) $$S^{\alpha }={\left(X^{\alpha }\right)}^{T}D^{\alpha }X^{\alpha }$$

where:

(18) $$D^{\alpha }=\mathrm{diag}\{\lambda_{1}^{\alpha },\lambda_{2}^{\alpha },\ldots ,\lambda_{m}^{\alpha }\},\lambda_{1}^{\alpha }\geq \lambda_{2}^{\alpha }\geq \ldots \geq \lambda_{m}^{\alpha }$$

and *X^α^* ∈ *_R_m*^×^*m* comprises the corresponding eigenvector. The optimum projective matrix $X_{\text{opt}}^{\alpha }$ comprises *d* (*d* ≤ *m*) eigenvectors corresponding to the largest eigenvalues. Thus, the reduced gait features $A_{k}^{\alpha }$ is:

(19) $$Y_{k}^{\alpha }=A_{k}^{\alpha }\times X_{\mathrm{opt}}^{\alpha },k=1\ldots N$$

The energy of 2DPCA is:

(20) $$E^{\alpha }=\sum_{i=1}^{d}\lambda_{i}^{\alpha }/\sum_{i=1}^{N}\lambda_{i}^{\alpha }$$

The value of *d* is selected according to *E*, where *E* denotes the information rate of reducing the dimensionality. It is usually around [0.9, 1]. In paper, *E* is set to 0.95 which gives a good reduction and recognition result. Since a CGCIs-DCT is used to represent gait features, each image has three matrices, one for the *R* component $A_{k}^{\alpha R}$, another for the G component $A_{k}^{\alpha G}$ and the third for the *B* component $A_{k}^{\alpha B}$. 2DPCA is performed on each of these matrices.

#### 4.2.4. Recognition

Each probe gait sequence is first processed to estimate their view *è* and generate the corresponding probe gait features. The gallery gait set that has the most similar view is selected for gait recognition.

2.5D probe gait features with *è* view are projected onto a plane to generate the CGCI and 2DPCA based DCT coefficients matrix denoted by $Y_{t}^{\theta }=\{Y_{t}^{\theta R},Y_{t}^{\theta G},Y_{t}^{\theta B}\}$, where the superscripts *R*, *G* and *B* respectively denote the *R*, *G* and *B* components. We define the Euclidean distance:

(21) $$D(Y_{t}^{\theta \mathrm{col}},Y_{k}^{\theta \mathrm{col}})=\sqrt{{\left|Y_{t}^{\theta \mathrm{col}}-Y_{k}^{\theta \mathrm{col}}\right|}^{2}},k=1,\ldots ,c$$

where $Y_{k}^{\theta }$ is a gait feature matrix belonging to the *k*th class, *col* = {*R,G,B*}, and *c* is the number of class in training samples. Denote *W_i_* as the ith class of training samples. The smallest distance is chosen as the recognition result, *i.e.*, $Y_{t}^{\theta \text{col}}\in W_{i}$ if:

(22) $$D(Y_{t}^{\theta \mathrm{col}},Y_{k}^{\theta \mathrm{col}})=\overset{c}{\underset{k=1}{\min}}\sqrt{{\left|Y_{t}^{\theta \mathrm{col}}-Y_{k}^{\theta \mathrm{col}}\right|}^{2}}$$

We define the final fused distance measure:

(23) $$D_{\mathrm{fused}}=k_{1}\cdot D(Y_{t}^{\theta R},Y_{k}^{\theta R})+k_{2}\cdot D(Y_{t}^{\theta G},Y_{k}^{\theta G})+k_{3}\cdot D(Y_{t}^{\theta B},Y_{k}^{\theta B})$$

where *k*_1_ = *k*_2_ = *k*_3_ = 1/3 Let $Y_{t}^{\theta }\in W_{i}$ if $D(Y_{t}^{\theta },Y_{k}^{\theta })=\min D_{\mathrm{fused}}$.

## 5. Experiments

### 5.1. 2.5D CSU Point Cloud Gait Database

**Figure 13.**
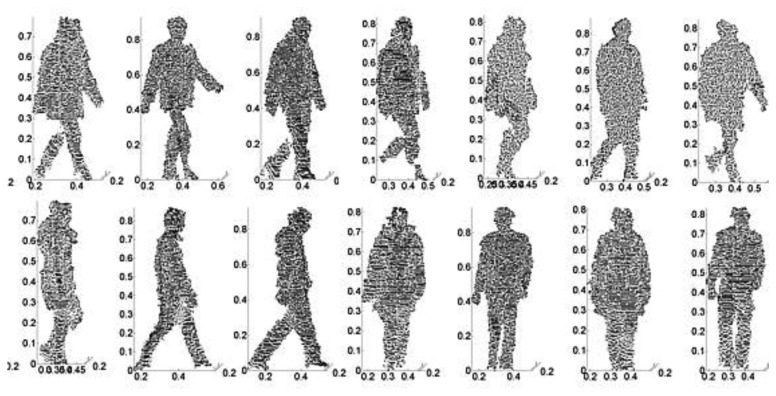
2.5D point cloud gait features with different views.

Hofmann *et al.* [24] presented a 2.5D TUM-GAID Database with depth information. However it is a gait database with depth information only. It is neither a 2.5D point gait database nor a multi-view database for gait. Since there are no publicly available 2.5D multi-view gait databases, we created the CSU database to evaluate the extraction of 2.5D gait features (e.g., as shown in Figure 13) and the identification algorithm using CGCI.

The database consists of 100 subjects, the data of each subject has 0°, 15°, 30°, 45°, 60°, 75°, 90° views. Each gait of the sample is captured three times. We use a single Microsoft Kinect camera to capture the videos. Each video sequence is of 8 s duration, recorded at full frame rate (30 frames/s). The original video format is 24-bit full-colour JPG and depth image files with resolution of 640 × 480. We extracted the subjects' data that have been segmented from background using OpenNI and generate 2.5D point cloud sets that contain the subjects' gait features.

### 5.2. Point Cloud Registration

**Figure 14.**
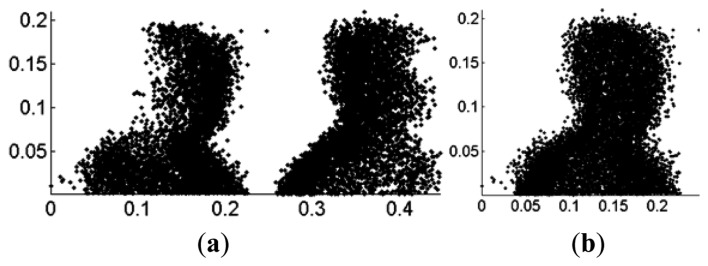
Registration result: (**a**) before registration; and (**b**) after registration.

**Figure 15.**
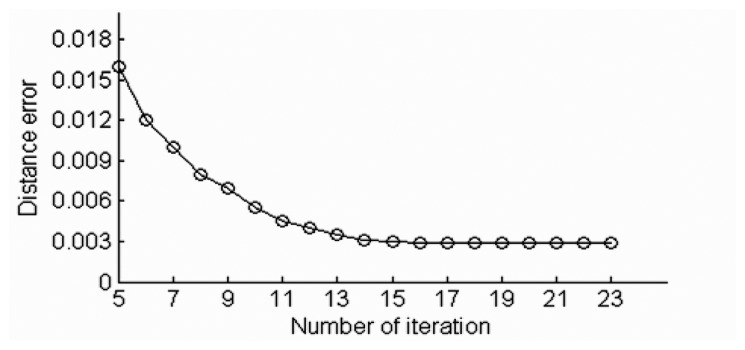
Relation between distance error and the number of iteration.

The point cloud registration algorithm using head point cloud data is proposed in Section 3.3. Object No. 8 in CSU Point Cloud Gait Database is taken as an example. The original views of mixed head point cloud data are 0° and 90°. Figure 14a shows the mixed head point cloud data in *β* = 45° after rotation from the original views. Figure 14b represents the data after registration with *θ_min_* = 45°, *θ_max_* = 90° and *β* = 45° in Equation (3). Figure 15 shows the relation between distance error *e_k_* and the number of iteration. We set the end condition for iteration as:

(24) $$e_{k}-e_{k-1}>ɛ,e_{k}=\sum_{i=1}^{\min(\text{m},\text{n})}{\left\|R_{k}l_{\theta_{\max}}^{i}+T_{k}-l_{\theta_{\min}}^{i}\right\|}^{2}$$

where *k* denotes the number of iteration, and *ε* = 10^−5^. Table 2 shows the point cloud registration result. Matrices *R* and *T* matrix are then used in full gait body to gain full registration gait model. By rotating the full registration gait model *S_â_* using Equation (5), the gait features for different views are obtained.

### 5.3. Multi-View Gait Recognition Experiments and Results

In order to evaluate the effectiveness of the proposed algorithm, experiments are carried out to compare the multiple-view gait recognition performances using three different methods. The first method uses the VTM technique and GEI as gait features [6]. The second method uses the 3D-based VTM technique and GEI as gait features [7]. It uses a 3D gait database comprising visual hulls with intact 360 degree body surfaces to construct the VTM model and realize view-invariant gait recognition by the VTM technique. In our experiment, we use our 2.5D voxel model which only has one side surface portion of the human body instead of the 3D data used in [7]. The third method uses our multi-view synthesizing method based on point cloud registration (MVSM).

The 2.5D CSU Point Cloud Gait Database is used in the experiments, where each gait sample is captured three times for each view. The database is divided into two sets. In all experiments, the two sets of gaits with different views are used. But only 0° and 90° views data from one set are retained for training and also as the gallery data sets for evaluating performance of multi-view gait recognition. The other set is used as the probe data set with different views.

Figure 10 illustrates the overview structure of the proposed gait recognition method. The three methods are trained using two standard reference gait view sets instead of the multi-view gait data in [6, 7]. The reference gait view *θ_min_* and *θ_max_* are set to 0° and 90°, respectively. The step interval Δ*θ* is set to 15°. Therefore, the VTM in VTM-GEI [6] method is constructed from GEI features with just 0° and 90° views from the training data set. The VTM in the 3D-based VTM-GEI [7] method is also constructed from GEI features but with more views. It includes 0° and 90° of the original views and others, *i.e.*, 15°, 30°, 45°, 60°, 75° views data that are generated by rotation from 0° and 90° (not from database). The GEI features are extracted from the B component in CGCI that indicates the average silhouette information of gait.

**Figure 16.**
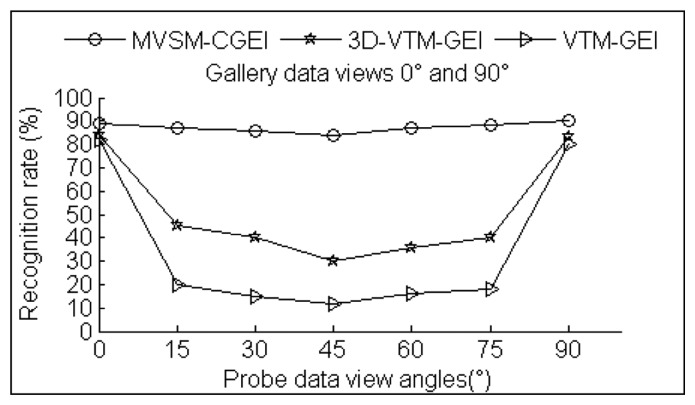
Comparison of recognition performance using different approaches.

To compute the comparison chart, in the VTM method the probe gait data from one view is transformed to a feature data under two other views that match one of the views (0° or 90°) in gallery gait database. In our proposed approach, the probe gait data from one view is matched with the corresponding synthesized galleries from (0° or 90°) data, and the gait similarity measurement is then calculated. Figure 16 shows the performances of the three methods.

**Figure 17.**
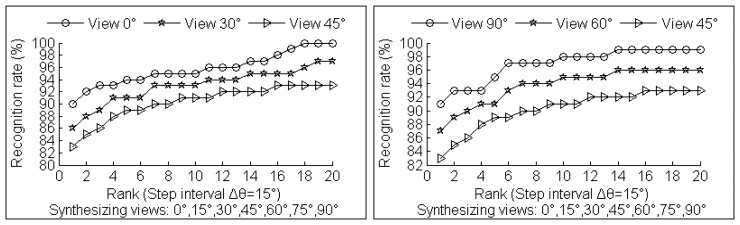
Recognition results with step interval Δ*θ* = 15°.

**Figure 18.**
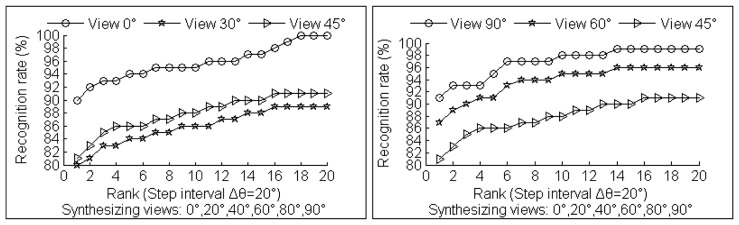
Recognition results with step interval Δ*θ* = 20°.

Additional experiments that set different step intervals in multi-view synthesizing process are conducted to show the improvement of the view-invariant 2.5D gait recognition. The results are shown in Figures 17 and 18. Cumulative Match Scores (CMS) are used to illustrate our 2.5D view-invariant gait recognition results. The CMS value α corresponding to rank r indicates a fraction 100·α% of probes whose top r matches must include the real identity matches.

Figure 16 shows that the proposed 2.5D view-invariant gait recognition approach outperforms the VTM method and the 3D-based VTM method. The 3D-based VTM method has better performance than the original VTM method because it can rotate the 2.5D gait data to extract multi-view gait features for VTM construction. The original VTM method has the worst performance, especially for the 30°, 45° and 60° views. This is because during training these views do not include the gallery sets that lead to bigger VTM angle transformation before the similarity measurement. The experimental results in [6–9] indicate the recognition rate degrades dramatically when the probe and gallery views differ by 30°. Another reason is that the number of multiple images from the gallery sets used in VTM construction and recognition directly influences its performance. In our method, only gallery data from two views are needed for training while three times more are used in [6–9]. Unlike the VTM-based method, our approach uses data registration and a 3D rotation method to synthesize different gallery data. As a result, five additional synthesized gallery data for views 15°, 30°, 45°, 60° and 75° are used that overcome the big angle transformation problem. This is the main reason why our method has achieved higher recognition results for probe views of 15°, 30°, 45°, 60° and 75° in Figure 6.

There are several other reasons why our method achieves significantly better performance. The first is that our multi-view 2.5D gait recognition method uses a single Kinect camera whereas VTM and other multi-view gait recognition approaches use multiple cameras or multi-view images. The experiment shows the method in [7] requires an intact gait 3D surface. Its recognition result drops significantly when our two standard reference gait view sets with only one side surface portion of the human body are used. This is because of the self-occlusion problem when using incomplete 3D sets to rotate the 3D gait models to construct VTM.

The second reason is that view-invariant 2.5D features are effectively extracted through Gaussian curvature, where Gaussian curvature is invariant to affine transformation. It is view-invariant under certain class of geometric transformation including rotation, scaling and shearing. This improves the performance of 2.5D gait feature extraction for multi-view gait recognition.

The last key point is that compared to GEI, our CGCI contains more surface information. The CGCI are gray images with curvature information instead of binary silhouette images used in GEI. Furthermore, Figures 17 and 18 show that the test gait view closer to reference training views or synthesized gallery views has significantly better recognition rate. Thus, in real applications, step interval Δ*θ* can be set to be small to gain more accuracy in arbitrary view gait recognition, but with a larger size for synthesizing gait databases.

## 6. Conclusions

In this paper, a 2.5D voxel gait model is constructed by point cloud data captured by a Kinect camera. In order to achieve multi-view gait recognition with one single Kinect camera, a point cloud registration method is proposed to synthesize different view gait features. Density of point cloud, Gaussian curvature and mean curvature are then introduced for extracting 2.5D gait features, which are projected to 2D RGB space to generate CGCI as expression of gait features.

The experimental results show that the proposed method is more effective than VTM-based multi-view gait recognition and other multi-view gait recognition methods using a single camera. Our 2.5D view invariant gait recognition based on point cloud registration approach needs only one camera and does not use multi-view images to achieve gait training and recognition. It achieves arbitrary view gait recognition without any camera calibration information. These advantages enable the proposed method to be used in many practical surveillance applications.

## Figures and Tables

**Table 1.** Gait view angle estimation results.

*θ* **View Angle of Test Data**	**Estimation Result***θ_est_*	

	*_Ē_*_[_*θ_est_*_]_	**σ^2^**
0°	0.55°	0.17
15°	15.62°	0.32
30°	29.47°	0.26
45°	44.31°	0.21
75°	74.29°	0.23
90°	90.48°	0.09

**Table 2.** Point Cloud Registration Result.

**Number of Iterations**	**Pixels**	***R* Matrix**	***T* Matrix**
18	10,062	$\left(\begin{array}{ccc} 0.998 & -0.002 & 0.329\\ 0 & 0.999 & 0.008\\ -0.352 & -0.007 & 0.991 \end{array}\right)$	$\left(\begin{array}{c} -0.2118\\ -0.0012\\ -0.0011 \end{array}\right)$
